# Social Protection and Foundational Cognitive Skills during Adolescence: Evidence from a Large Public Works Program

**DOI:** 10.1093/wber/lhad035

**Published:** 2023-11-13

**Authors:** Richard Freund, Marta Favara, Catherine Porter, Jere Behrman

**Affiliations:** University of Cape Town School of Economics, Cape Town, South Africa, 7701; Research Affiliate at the Oxford Department of International Development; Oxford Department of International Development, University of Oxford, Oxford, UK, OX1 3TB; Director of Research at Young Lives; Director of Young Lives; Development Economics at the Lancaster University Management School, Lancaster, UK, LA1 4YX; University of Pennsylvania School of Arts and Science, Philadelphia, USA, PA 19104

**Keywords:** foundational cognitive skills, public works programs, Ethiopia, PSNP, executive function

## Abstract

Many low- and middle-income countries have introduced public works programs (PWPs) to fight poverty. This paper provides the first evidence that children from families who benefit from PWPs show increased foundational cognitive skills. The results, based on unique tablet-based data collected as part of a long-standing longitudinal survey, show positive associations between participation in the Productive Safety Net Programme (PSNP) in Ethiopia during childhood with long-term memory and implicit learning, and suggestive evidence for working memory. These associations appear to be strongest for children whose households were still PSNP participants in the year of data collection. Evidence suggests that the association with implicit learning may be operating partially through children's time reallocation away from unpaid labor responsibilities, while the association with long-term memory may in part be due to the program's success in remediating nutritional deficits caused by early-life rainfall shocks.

## 1. Introduction

Social protection programs to combat poverty are now widespread around the world (International Labour Organization 2021). In Africa alone, the number of social protection programs almost tripled in the first 15 years of the twenty-first century (Cirillo and Tebaldi 2016) to the extent that, today, all African countries operate at least one such program (Beegle, Honorati, and Monsalve 2018). Many low- and middle-income countries (LMICs) have introduced public works programs (PWPs) as one such form of social protection program for fighting poverty and to provide social safety nets in light of different types of income shocks generated by weather (e.g., Ethiopia, India, Malawi, South Africa), rising prices (e.g., Argentina, India, Mexico), and conflict and political instability (e.g., Comoros, Côte d'Ivoire, Sierra Leone) (Subbarao et al. 2012). These programs provide temporary cash-for-work opportunities to boost poor households’ incomes and to develop infrastructure for local communities.

While PWPs do not target children directly, they may affect children's development through two primary, and possibly contradictory, mechanisms working through participating households (Woldehanna 2010). First, by increasing household income, PWPs may positively impact cognition through an increase in food consumption and nutrition (Behrman 1996; Glewwe, Jacoby, and King 2001; Maluccio et al. 2009). Second, given PWPs’ adult work requirements, they may alter parents’ and children's time use, affecting the children's cognitive development. For example, adults may reduce their time caring for and interacting with children in order to work more, and children may substitute for adult labor in family businesses and reduce time learning (Basu and Van 1998). Through these channels, PWPs may affect lifelong learning opportunities for such children, which has been advocated as a necessary condition for allowing future generations to escape poverty, as expressed in the United Nations Sustainable Development Goals (SDGs).

A small number of previous studies investigate the impacts of PWPs on child development in LMICs. Evidence from the largest PWP in the world, India's Mahatma Gandhi National Rural Employment Guarantee Scheme, is mixed (Mani et al. 2020; Shah and Steinberg 2021). For the Ethiopian Productive Safety Net Programme (PSNP), Favara, Porter, and Woldehanna (2019) found significant positive associations with both numeracy and vocabulary. However, a major limitation of the existing literature concerning the effect of PWPs, and of social programs more generally, on skills development in LMICs is that cognitive skills are measured using domain-specific cognitive achievement test scores (e.g., test scores in mathematics, reading comprehension, and vocabulary knowledge) rather than foundational cognitive skills (FCS).^1^ A deeper understanding of how PWPs and other policy interventions can mitigate the effects of poverty on the formation of FCS is still needed to complete the picture.

This paper contributes to filling this gap by investigating the associations of the Ethiopian PSNP, the largest PWP and the second-largest social protection program in Africa, with the development of FCS for a cohort of children tracked since infancy through adolescence. FCS are linked to children's subsequent capabilities, learning, productivities, and welfare. A substantial body of research in high-income countries has linked cognitive function measured in laboratory settings to real-world behaviors, demonstrating that individual differences in FCS successfully predict educational and labor-market outcomes (Blair 2002; Heckman, Stixrud, and Urzua 2006; Blair and Razza 2007). An increasing body of evidence also indicates that FCS may increase in response to investments of time and effort by parents and teachers, suggesting that FCS, unlike many other developmental and cognitive processes, remain malleable into late stages of childhood and adolescence (Diamond et al. 2007; Jaeggi et al. 2008; Holmes, Gathercole, and Dunning 2009).

Furthermore, family and environmental impacts on FCS precede school performance deficits related to poverty, and predict subsequent schooling outcomes (e.g., math-test performance) over and above current schooling outcomes (Blair and Razza 2007). Policy interventions that are able to mitigate the effects of early poverty on FCS formation could thus improve children's current schooling and also their potential future outcomes. Altering FCS may be one of the few means available for mitigating the adverse effects of early childhood poverty, undernutrition, and inadequate education on cognitive skills among older children.

Despite this, there is no population-based evidence from LMICs; available evidence is from small samples in high-income countries. This paper improves understanding of how policy interventions can attenuate the effects of early-life deprivations and promote lifelong learning opportunities for all (UN Sustainable Development Goal #4). Evaluation of the PSNP is of general interest, given that the program is implemented at scale, using governmental systems, in a populous low-income country in Africa.

This paper uses unique data on four FCS measures (long-term memory, inhibitory control, working memory, and implicit learning) collected in Ethiopia as part of the Young Lives Study (YLS), a longitudinal study following the same children since 2002. To mitigate concerns about bias due to household selection based on unobserved variables, a restricted comparison sample that is similar to PSNP recipients is constructed. This study finds positive associations of the PSNP with long-term memory and implicit learning, and weaker evidence for working memory. The associations between the PSNP and long-term memory are significantly larger for females. There are no significant associations of the PSNP with inhibitory control. Comparison of the results to domain-specific vocabulary and mathematics tests highlights the importance of considering FCS for understanding the full effect of the PSNP on children's cognition.

This paper also investigates which mechanisms help explain any significant associations of the PSNP with FCS. It finds suggestive evidence that, in part, the association with implicit learning may reflect children's time reallocation away from unpaid working responsibilities, while the association with long-term memory may be due to the program's success in remediating early nutritional deficits. Lastly, given the link to early nutrition, the paper explores the remediation channel further by using an exogenous source of variation: early-life rainfall shocks. To do so, Young Lives data is matched with gridded data on monthly precipitation to generate community-level rainfall estimates. This study finds evidence that the positive associations of the PSNP with long-term memory are driven by children who experienced at least one rainfall shock in their first year of life.

This paper offers two main contributions. The first is the use of unique data on FCS measured through a novel touch-screen tablet application, collected as part of a large cohort study in LMICs. Unlike most papers considering the effects of PWPs and other social-protection programs on cognitive skills, the measures in this paper are foundational to a wide range of learning and are not domain-specific; they should, therefore, be relatively free of bias due to the language of implementation or differences in the children's, caregivers’, or communities’ beliefs in the value of academic knowledge.^2^ Second, this is the first study to examine the remediation role of a PWP on multiple FCS in a LMIC setting.

The rest of this article is structured as follows: section 2 provides a brief outline of the PSNP structure and background. Section 3 outlines the data used, while section 4 presents the estimation strategy used. The results are reported in section 5, while potential mechanisms are explored in section 6. Section 7 concludes.

## 2. The PSNP in Ethiopia

The PSNP was introduced in Ethiopia in 2005 as a national rural safety-net program. Its objective is to provide transfers to the food-insecure population in chronically food-insecure *woredas* (districts),^3^ as well as to assist households when food production and other sources of income are insufficient (Ministry of Agriculture and Rural Development 2004). The PSNP, being centrally co-ordinated by the Government of Ethiopia, represented a change from emergency food-for-work programs provided on an irregular basis by different parties (Porter and Goyal 2016). The PSNP was specifically conceived as a multiyear program to provide regular and reliable transfers over several years in a way that prevents household asset depletion and creates community assets (Sabates-Wheeler et al. 2021).

The PSNP operates as a safety-net mechanism, whereby the transfers benefit poor rural households mainly through public-works participation (80 percent), with a small proportion of households receiving unconditional direct support (food and/or cash transfers) in the absence of available adult labor in the household. As part of the program, PSNP beneficiaries are eligible to work five days per household member (aged 18–60) per month (Sharp, Browne, and Teshome 2006). While the program started with 4.5 million beneficiaries in 2005, in 2013–the year of this study—the PSNP supported 7.2 million people (roughly 8 percent of the national population) in 290 chronically food-insecure *woredas* (Favara et al. 2019).^4^

Despite a cash-first principle, only 15 percent received cash exclusively in 2012/13; 18 percent received food only and 67 percent received a mixture of cash and food. In 2010/11, median transfer values were just under 500 birr (roughly £20) per year for a household of five, equivalent to approximately 13 percent of the value of the poverty line (DFID 2013). The goal is that the program should improve household food security up to the point that the household graduates: “A household has graduated when, in the absence of receiving PSNP transfers, it can meet its food needs for all 12 months and is able to withstand modest shocks” (FSCB 2007). In practice, households were nominated for graduation based on annual socioeconomic assessments on household assets, alternative sources of income, and agricultural production and livestock (Devereux et al. 2014). If households were deemed to have reached the benchmark, they were considered ready to graduate. Between 2005 and 2014, approximately 500,000 beneficiaries graduated from the program (Hoddinott 2014). In the years 2009–2013, graduation benchmarks were different across communities, unclear to participants, and often households felt they had graduated prematurely, due to pressure to meet targeting requirements (Devereux et al. 2014).

The PSNP previously has been found to be effective in improving household food security, consumption, and children's nutritional status. Berhane et al. (2011) found significant positive effects of the PSNP on household food security and consumption status, and Berhane et al. (2014) observed significant improvements in food security for households that participated in the program for more than four years. Alderman and Yemtsov (2012) concluded that PSNP-recipient households avoided selling assets and using savings to buy food in times of food shortages, and Porter and Goyal (2016) found that the program led to important medium-term nutritional impacts for children at different ages (from age 3 to 15).

While the impact of the PSNP on household consumption and food insecurity is well established, the work requirement of the program means that there could be an ambiguous effect on children's outcomes, including school enrolment and cognitive development, through its effect on the time-use allocation of adults and children living in the households (Favara et al. 2019). If, for example, child labor acts as a substitute for adult labor on the family farm/enterprise or in domestic tasks, this substitution could offset the (positive) income effect of the program, resulting in a potential worsening of children's outcomes. Additionally, if time spent with parents has a positive impact on cognitive outcomes (e.g., Sheridan and McLaughlin 2016), an increase in parental time spent at work could have adverse effects.

While the minimum age for PWP participation is 18 years, according to Sharp, Brown, and Teshome (2006) approximately 8 percent of the PSNP workers were under 18. Tafere and Woldehanna (2012) found negative effects of the PSNP on children's time use, arguing that the program increased time spent on both paid and unpaid work among adolescents. The authors note that the PWP work requirement led households to supplement adult labor with child labor. There is also evidence that time-use implications may differ according to the child's gender. Camfield (2014) found evidence of girls working directly in the PSNP program or increasing their household tasks in response to caregivers’ participation in the program. Hoddinott et al. (2010) found that participation in the PSNP led to a reduction in time spent on agricultural labor among boys aged 6–16, and that younger boys aged 6–10 as well as older girls aged 11–16 spent less time on household tasks. However, girls younger than 11 spent more time on tasks within the household and reduced their school enrolment.

The theoretical net effect of the program on cognitive skills is thus ambiguous. Participation in the PSNP would be expected to have a positive effect on skills development of participant household children if the positive income effect on nutrition outweighs any negative time-use effects (Behrman 1996). However, if labor-supply demands on adults change children's time uses, there may be harmful time-use effects and negative effects on children's cognitive skills (Basu and Van 1998). Favara et al. (2019) estimated the impact of the PSNP on children's domain-specific learning outcomes (measured through test scores) and found a small but significant positive effect of the program on both numeracy and vocabulary, suggesting that the positive income effect may be dominating any negative substitution effects.

## 3. Data

This paper uses two datasets for its main analysis: the Young Lives survey in Ethiopia and data that were collected on FCS during the fourth round of the Young Lives survey.

### Young Lives Data

The YLS Ethiopia is a longitudinal study initiated in 2001 to investigate the changing nature of childhood poverty in Ethiopia (Favara et al. 2021). The first survey took place in 2002, with four further in-person rounds of data collection in 2006/7 (Round 2), 2009/10 (Round 3), 2013/14 (Round 4), and 2015/16 (Round 5). The cohort children were aged 6–18 months in 2002. The RACER tests of FCS were administered in Round 4, when the children were 11–12 years old.

The initial 2002 survey collected information on 1,999 children. These children were selected from 20 *woredas* in the states of Amhara, Oromia, the Southern Nations, Nationalities and People's Region (SNNP), Tigray, and Addis Ababa. The *woredas* were picked in an attempt to oversample areas with food-deficit status, capture ethnic and geographic diversity, and find urban/rural and development-level balance (Outes-Leon and Sánchez 2008). Hence, Young Lives is not a nationally representative survey; comparison to national statistics data indicate that Young Lives households are generally poorer than the average Ethiopian household. Despite this, existing research has found that the Young Lives sample covers the diversity of children in the country in a wide variety of attributes and experiences (Outes-Leon and Sánchez 2008). Within each *woreda*, a village was randomly selected, and households were randomly contacted (moving clockwise from a random initial location) until approximately 100 eligible families were found. The study managed to keep attrition rates low, compared to other longitudinal studies: after four survey rounds, only 6.8 percent of the 2002 rural sample was lost.^5^

In all rounds, three main questionnaires were administered to capture various characteristics that are expected to influence the status of the children: a child questionnaire with data on health, anthropometrics, and individual characteristics; a household questionnaire including data on caregiver background, livelihood, household composition, socioeconomic status, and shocks; and a community questionnaire containing information on demographic, geographic and environmental characteristics, social environment, infrastructure, the economy, health and education.

**Figure 1.**
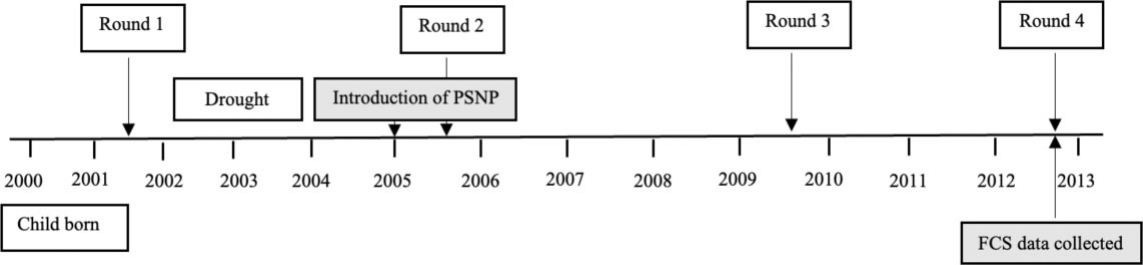
Timeline of the Productive Safety Net Programme (PSNP) Introduction and Data Collection. *Source*: Authors' own illustration. *Notes*: Figure shows the timing of the first four Young Lives survey rounds, as well as the foundational cognitive skills data collection.

In 2013, the PSNP was operating in 14 YLS *woredas*, with approximately 21 percent of the Round 4 sample (398 out of the 1,873 households) being active beneficiaries of the program. Households were asked whether they had received payments from public works or direct support within the PSNP framework in 2006, 2009, and 2013. They were also asked in which years they were enrolled in the PSNP, how much they had received in the past 12 months (cash or in-kind payment),^6^ and whether, to their knowledge, they had been shortlisted for the program or had graduated from the program. Figure 1 shows the timing of the first four YLS rounds as well as the introduction of the PSNP.

### Rapid Assessment of Cognitive and Emotional Regulation (RACER)

Data on FCS were obtained during the fourth YLS survey round. FCS were measured using a series of tablet-based tasks through RACER (Rapid Assessment of Cognitive and Emotional Regulation) (Hamoudi and Sheridan 2015; Ford et al. 2019). RACER is a novel touch-screen tablet application that uses five short tasks (one to four minutes each) to assess four components of FCS in children aged six years and older (and adults). This section briefly describes the FCS that were assessed using RACER tasks, and the measures used in the empirical analysis. More information about RACER and the FCS measured in the YLS can be found in Behrman et al. (2022).

RACER measures four FCS: Long-term Memory (LTM), Inhibitory Control (IC), Working Memory (WM), and Implicit Learning (IL). In each cognitive task, two measures are identified: the challenge measure and the baseline measure. Challenge measures aim to assess explicitly an individual's FCS. Baseline measures are identical to challenge measures—in terms of general concentration, visual input, and motor response—but lack the specific manipulation that requires an individual to employ FCS to get the trial correct (Ford et al. 2019). A respondent's performance on both baseline and challenge measures depends on many skills and competencies, including familiarity with touch screen tablets or computers (Hamoudi and Sheridan 2015).^7^ During analysis, FCS challenge measures are therefore always compared to baseline measures so that the within-person difference is used as an indication of the person's FCS ability specifically, rather than their general ability to perform cognitive tasks or use electronics. Below is a brief explanation of how each task is performed and fig. S1.1 in the supplementary online appendix (available at *The World Bank Economic Review* website) shows how the tasks are presented to the child.

In Ethiopia, the RACER data were collected for 1,809 children (aged 11–12 years old at the time). Administration time ranged between 30 and 45 minutes. RACER was administered to 97 percent of the children available for interviews, with an attrition rate considerably smaller than for other cognitive test scores administered.^8^ For the analysis in this paper, each component of FCS is defined so that higher values are better (see Behrman et al. 2022 for more details).

*Long-term Memory*. LTM is the ability to encode, retain, and retrieve new knowledge. It supports the capacity to acquire new knowledge and learn from experience. In each trial of the LTM game, participants are shown a figure and must choose the correct matching figure out of four options. Participants are presented with a total of 20 figures, 12 of which are grouped arbitrarily into 6 pairs. For each trial, respondents see one member of a pair at the top of the screen, and at the bottom they are presented with four choices, consisting of the other shape in the pair plus three incorrect lures. Respondents see each pair a total of four times; in the first six trials (baseline trials), each pair is encountered for the first time, whereas in the second, third, and fourth cycles of six challenge trials the respondent might be able to remember each pair. In both cases, performance is measured as the average percentage of correct answers at the first touch. Tests of this kind are dependent on the function of the hippocampus in both children and adults (Bechara et al. 1995). The hippocampus is susceptible to the effects of chronic stress, which is one reason that poverty might impact performance on this task (McEwen 2001; Farah et al. 2006; Shonkoff, Boyce, and McEwen 2009; Hanson et al. 2011). Long-term memory is necessary for the acquisition of explicit knowledge in school and other settings.

*Inhibitory Control*. IC is the ability to override counterproductive impulses and resist distraction by irrelevant information. Assessed skill of inhibitory control has been observed to correlate with impulse control in and out of the classroom (Barkley, Grodzinsky, and DePaul 1992). In the RACER application, participants are presented with differently colored and patterned circles on either side of the tablet screen and must touch the center, or opposite, side of the screen as quickly as possible depending on whether a solid or striped circle appears. Specifically, either a solid yellow or striped pink circle is presented one at a time on either the right or left side of the tablet screen. Respondents are told that when a solid yellow circle appears, they should touch the center of the circle as quickly as possible (same-side trials). However, when a striped circle appears, they should touch the opposite side of the screen from the stimulus as quickly as possible (opposite-side trials). For all trials, performance is assessed as an equally weighted average of the inverse of response time (in milliseconds) and the inverse of the logarithm of the (Euclidean) distance (in pixels) from each touch to the nearest stimulus. The measures from the same-side trials and opposite-side trials correspond to the baseline and challenge trials, respectively.

*Working Memory*. WM refers to the ability to hold in mind and manipulate information that is no longer present in the environment. While this is a simple cognitive function, it is a necessary component of many more complex abilities such as high-level reasoning, planning, or language comprehension (Miyake et al. 2001). Children perform better on working-memory tasks as they get older, and both children and adults recruit the prefrontal cortex when performing working-memory tasks (Thomason et al. 2009). Working-memory ability in childhood is linked with performance in school even after controlling for content of knowledge (Blair 2002), and training of working memory and executive function more generally is associated with decreased behavioral problems and increased academic performance (Diamond et al. 2007; Klingberg 2010).

RACER measures WM by presenting participants with one to three dots on the tablet screen for a limited time, and then asking participants to touch the screen as close as possible to where the dots were. Thus, WM is measured using a spatial delayed-match-to-sample task. Specifically, for each trial, respondents are shown one, two, or three blue dots on the screen, which remain for two seconds. After this, the screen goes blank for either 0.1 seconds (short delay) or three seconds (long delay). After the delay, participants are asked to touch the screen as close as they can to where the dots used to be. Baseline trials are those with short delays and one dot. Challenge trials are those with long delays and multiple dots (two or three). In both measures, performance is assessed as the inverse of the logarithm of the (Euclidean) distance (in pixels) from each touch to the nearest stimulus.

*Implicit Learning*. IL is the ability to learn without conscious awareness (sometimes described as “muscle memory”). This ability is a very basic and primary form of learning, as it relies on the basal ganglia, deep brain structures that are conserved across species (Aron, Gluck, and Poldrack 2006). To measure IL, the RACER presents participants with 175 dots (one at time) to touch as quickly as possible, with some successive dots following no pattern, and others following repeated cycles of locations. Specifically, a total of 175 identical stimuli are presented one at a time in one of four screen locations (roughly corresponding to the top left, top right, bottom right, and bottom left of the screen). Each stimulus remains on the screen for up to 1 second; the respondent's task is to touch the stimulus before it disappears. The succession of screen locations follows no pattern for the first 35 stimuli (Block 1), but then the next 70 stimuli (Block 2 and 3) are presented in 10 repeated cycles of 7 locations, and then the next 35 again follow no pattern (Block 4), and the last 35 (Block 5) are 5 more repeats of the pattern observed for the first time in block 2. IL is assessed by measuring the inverse of the average response times (in milliseconds) during the patterned blocks (challenge trials), using the analogous performance in nonpatterned blocks (baseline trials). In the game described above, individuals press more quickly when the movement of the dot follows a pattern, even when they themselves are unaware of the pattern (Pasupathy and Miller 2005). The ability to speed-up with patterned presentation relative to random presentation, implicit learning, has been strongly linked with language acquisition in infancy and early childhood (Arnon 2019).

## 4. Methodology

This paper aims to estimate whether access to the PSNP is associated with improved FCS among children from disadvantaged backgrounds. The main empirical concern when estimating the effects of the PSNP on FCS is that of endogenous program placement (at both the geographic and household levels). Since access was not randomized, this leads to challenges in finding a convincing identification strategy for assessing the impact of the program. Before the introduction of the program, PSNP beneficiary households in the YLS sample tended to be poorer and had lower average per capita expenditures (table S.1.1 in the supplementary online appendix). Thus, a naïve comparison of children in PSNP households with those in nontreated households would likely bias any estimated treatment effects downwards.

This section describes the sample used for the empirical analysis and the empirical approach adopted to mitigate such concerns.

### Treatment and Control Groups

The regression sample is limited only to children who were living in rural areas in 2006, since the PSNP is a rural program. Children living in households that only started receiving the PSNP in 2012—just before the survey rounds where FCS are measured—were also dropped. Lastly, as the empirical strategy utilizes community fixed effects, only *woredas* in which the PSNP was operating in 2013 (14 *woredas*) were included. Households are defined as treated if they answered *yes* to the question on PSNP participation (either food- or cash-for-work, or direct transfers of food or cash).^9^

To mitigate concerns related to systematic differences between the control and treatment groups due to endogenous allocation of the PSNP program, following Favara et al. (2019) and Porter and Goyal (2016) this paper constructs a “restricted” comparison group to use in the empirical analysis that is arguably more comparable to PSNP beneficiary households. This only includes: (1) those who received any kind of governmental program (food-for-work, cash-for-work, food aid) in 2006 since they were in some sense eligible for PSNP, and therefore likely quite similar to eventually treated households; and/or (2) households who reported in 2009 that they had been shortlisted for PSNP, because while community-level shortlists were drawn up, some households did not receive PSNP due to budget allocations not being sufficient from the next-higher level of administration (Favara et al. 2019).^10^

Panel A in table 1 compares pre-program (2006)^11^ characteristics of the sample of children in PSNP beneficiary households to children in the restricted control group sample. It reports no significant differences in the average age, gender, household size, monthly food expenditure, wealth index,^12^ and livestock ownership between the groups of PSNP and restricted control children. Table 1 also finds no difference in parental employment, access to services, whether a household has an influential connection in their village, the number of people a household can rely on in times of financial need, and whether the primary caregiver believes that a 12-year old child in their village should leave school to go work if the household needs extra income.^13^

**Table 1.** Descriptive Statistics

Variable	PSNP	Control
	(1)	(2)
**Panel A. Background characteristics (2006)**		
Child's age (in years)	4.65	4.73
	(0.48)	(0.46)
Male child	0.54	0.55
	(0.50)	(0.50)
Household size	6.32	6.39
	(1.84)	(1.88)
Head of house schooling grade	1.33	2.09***
	(2.16)	(2.79)
Male head of house	0.84	0.96***
	(0.37)	(0.19)
Monthly food expenditure (birr)	61.28	64.14
	(44.03)	(42.42)
Wealth index	0.19	0.19
	(0.11)	(0.11)
Owns livestock	0.91	0.96
	(0.29)	(0.19)
Owns land of house	0.91	0.97**
	(0.28)	(0.16)
Owns house	0.93	0.95
	(0.25)	(0.23)
**Panel B. RACER outcomes (2013)**		
Long-term memory	0.01	−0.02
	(1.12)	(0.99)
Inhibitory control	−0.19	0.01***
	(0.69)	(0.72)
Working memory	0.03	0.02
	(1.01)	(0.97)
Implicit learning	−0.02	0.00
	(1.00)	(1.00)
Long-term memory (baseline)	−0.08	−0.02
	(0.99)	(1.00)
Inhibitory control (baseline)	−0.10	0.02*
	(0.70)	(0.68)
Working memory (baseline)	−0.00	−0.00
	(1.04)	(1.00)
Implicit learning (baseline)	−0.03	0.01
	(0.97)	(1.02)
*Number of children (full sample)*	422	187

*Source*: Data comes from the Young Lives Study (YLS) in Ethiopia. Data on foundational cognitive skills (FCS) were obtained during the fourth YLS survey round in 2013.

*Note*: Panel A reports the summary statistics of the background (pre-program) characteristics of the survey respondents. Wealth terciles are based on the Young Lives wealth index (Briones 2017). Panel B reports the summary statistics of the FCS variables. FCS scores are standardized, according to the distributions for the non-PSNP children. Columns (1) and (2) report the mean and standard deviation (in parentheses). Asterisks reflect *p-*values for *t-*tests for differences in means between children in PSNP households and control households.

***Significant at 1 percent. **Significant at 5 percent. *Significant at 10 percent.

However, the table indicates that nonrecipient control households are less likely to have a female head of household, have slightly higher average levels of schooling among the head of household, and are more likely to own the land that their house is on. These imbalances suggest that, if anything, PSNP children are more likely to come from relatively poorer households than their counterparts in non-recipient households. Given that FCS are typically positively associated with household wealth (Farah et al. 2006; Noble, McCandliss, and Farah 2007; Hackman and Farah 2009), this would imply that, if there is remaining selection bias on unobserved variables, this paper would be likely to *underestimate* the magnitude of the association between the PSNP and improved FCS.

Panel B in table 1 compares the standardized RACER scores (both the challenge and baseline measures) for all children in the PSNP and restricted control groups. It reports no significant difference in the FCS measures, except the inhibitory control measures, in which PSNP recipient households perform relatively worse than those from the restricted control group. Table S.1.3 in the supplementary online appendix provides information on a number of intermediate outcomes potentially linking enrolment in the program to changes in FCS. It reports no significant differences in pre-program nutritional status (Body Mass Index and the prevalence of stunting) and time-use categories. However, children in the restricted control group were relatively more likely to experience at least one rainfall shock during their first 1,000 days of life.

### Empirical Strategy

The primary specification used to test the association between the PSNP and FCS is as follows:


(1) \begin{eqnarray*}
Challeng{e}_{ihjk} = {\alpha }_0 + \delta PSN{P}_{hj} + {\beta }_1Baselin{e}_{ihjk} + {\beta }_2{{\boldsymbol{X}}}_{{\boldsymbol{hj}}} + {\beta }_3{{\boldsymbol{G}}}_{{\boldsymbol{ihjk}}} + {\gamma }_j + {\mu }_{ihjk},
\end{eqnarray*}

where the dependent variable *Challenge* is a variable used to denote the FCS challenge measurement of child *i* in household *h* in community *j* in RACER task *k*, and *Baseline* denotes the analogous baseline performance measurement. All FCS challenge and baseline measures are standardized by the control group means and standard deviations. On the right side of the equation, *PSNP* is the treatment variable identifying PSNP beneficiary households; ${\boldsymbol{X}}$ and ${\boldsymbol{G}}$ are vectors of household- and child-level characteristics, and task controls respectively. ${\gamma }_j$ represents community (unobserved) fixed effects, intended to capture community effects that may, for example, affect PSNP delivery and formation of FCS. ${\mu }_{ihjk}$ represents a mean-zero, idiosyncratic error. The coefficient of interest, $\delta $, denotes the association between the PSNP and FCS development. Standard errors are clustered at the *woreda* level, the unit of treatment assignment (Abadie et al. 2023), and *p-*values from wild-cluster-bootstrap standard errors are also presented due to the small number of clusters (Cameron, Gelbach, and Miller 2008). To deal with the potential issue of multiple hypotheses testing, sharpened *q*-values of Benjamini, Krieger, and Yekutieli (2006) are also reported for the main results.

This paper also investigates heterogeneous associations between the PSNP and FCS according to program graduation status and gender. The measurement of FCS comes shortly after the introduction of graduation from the PSNP, and it is therefore of interest to assess whether the associations are driven by those still receiving the program, or whether there are persistent associations among recently graduated participants. Regarding gender, on the one hand, son preferences may lead to greater impacts of the program for boys than for girls (Behrman 1988). On the other hand, however, girls may be treated as “luxury goods” in which investments in girls are very low for very poor households but increase relatively rapidly when income increases (Behrman and Deolalikar 1990).


(2) \begin{eqnarray*}
Challeng{e}_{ihjk} = {\alpha }_0 + \delta PSN{P}_{hj} + \gamma Mal{e}_{ihj} + \varphi PSN{P}_{hj}* Mal{e}_{ihj} + {\beta }_1Baselin{e}_{ihjk} + {\beta }_2{{\boldsymbol{X}}}_{{\boldsymbol{hj}}} + {\beta }_3{{\boldsymbol{G}}}_{{\boldsymbol{ihjk}}} + {\gamma }_j + {\mu }_{ihjk},\\
\end{eqnarray*}

All specifications control for the age and gender of the child, the child's main language, religion, and ethnicity, the socioeconomic status of the household (as measured through the YLS wealth index), the household size, the household head's gender and education (the highest schooling grade achieved by the household head), whether the household owned their house and the land on which the house was built, the household's food expenditure, whether the household has access to a school, whether the household has an influential connection, the number of people the family can rely on for financial need, and whether the caregiver believes that a child should leave school for work if needed. All variables were measured before the introduction of the program (in 2006). Control variables also include the weekday and the time of day when the FCS tasks were administered.^14^

## 5. Results

Table 2 reports the associations between each of the FCS measures and the PSNP (equation 1).^15^ The findings suggest that PSNP beneficiaries have significantly higher LTM and IL scores; on average, being a PSNP beneficiary is associated with an increase in the LTM and IL tasks of 0.21 and 0.12 standard deviations, respectively.^16^ The table does not report any significant associations between PSNP status and IC and WM.^17^, ^18^

**Table 2.** Associations between the Productive Safety Net Program and Foundational Cognitive Skills

	Foundational cognitive skills				Domain-specific cognitive skills	
	(1)	(2)	(3)	(4)	(5)	(6)
	LTM	IC	WM	IL	PPVT	Maths
PSNP ($\delta $)	0.211**	−0.019	0.070	0.123**	0.071	18.172**
	(0.078)	(0.059)	(0.058)	(0.050)	(0.084)	(7.415)
	{0.031}	{0.507}	{0.202}	{0.031}	{0.327}	{0.031}
Controls	Yes	Yes	Yes	Yes	Yes	Yes
${R}^2$	0.250	0.426	0.305	0.746	0.505	0.159
**By graduation status**						
2009-only PSNP beneficiaries	0.175*	−0.060	0.020	0.092*	0.132	26.346**
(graduated)	(0.093)	(0.068)	(0.062)	(0.046)	(0.089)	(8.199)
2009 & 2013 PSNP beneficiaries	0.234**	0.007	0.103*	0.143*	0.021	12.790
	(0.106)	(0.066)	(0.061)	(0.068)	(0.095)	(7.990)
Observations	608	609	608	608	527	609

*Source*: Authors’ analysis based on data from the Young Lives Study (YLS) in Ethiopia. Data on foundational cognitive skills (FCS) were obtained during the fourth YLS survey round in 2013.

*Note*: The top panel reports the associations between participation in the Productive Safety Net Programme (PSNP) and FCS, using equation (1). The table reports the OLS estimates with standard errors (reported in parentheses) clustered at community level. LTM = long-term memory, IC = inhibitory control, WM = working memory, IL = implicit learning. RACER outcomes are standardized using the means and standard deviations of the control group. Each coefficient comes from a different estimation of equation (1) for each outcome. All estimations include community fixed effects, and control for the age and gender of the child, the child's main language, religion, and ethnicity, the socioeconomic status of the household, the household size, the household head's gender and education, whether the household owned their house and the land on which the house was built, the household's food expenditure, whether the household has access to a school, whether the household has an influential connection, the number of people the family can rely on for financial need, and whether the caregiver believes that a child should leave school for work if needed. Columns (1) to (4) also control for the weekday and the time of the day when the FCS tasks were administered. All controls are measured in 2006. The lower sample size for the Peabody Picture Vocabulary Test (PPVT) is due to the fact that the test was not administered if the child's language was not Amharic, Oromifa, or Tigrinya. Domain-specific cognitive scores are standardized using Item Response Theory (Leon 2020). *p-*values are calculated using wild bootstrap standard errors (clustered at the community level) derived from running 1,000 replications. *q*-values are obtained using the sharpened procedure of (Benjamini, Krieger, and Yekutieli 2006), and are shown in curly brackets below the standard errors.

***Significant at 1 percent. **Significant at 5 percent. *Significant at 10 percent.

As a comparison, table 2 also reports the associations between participation in the PSNP and the Peabody Picture Vocabulary Test (PPVT), a widely-used test of receptive vocabulary (Dunn and Dunn 1997), and a mathematics test that was developed by the Young Lives survey team (Cueto and Leon 2012).^19^ The results suggest being a PSNP beneficiary is significantly associated with improved mathematics skills, but not language ability. Consequently, focusing only on domain-specific tests might lead one to conclude that the PSNP can only influence mathematical ability, but not other cognitive skills. However, the results in table 2 provide suggestive evidence that this is not the case; the PSNP may be able to affect broader, domain-general foundational cognitional skills through its potential effects on LTM and IL.

### Heterogeneous Associations by PSNP Graduation Status

The results in table 2 bundle the effect of the PSNP among both households who received the PSNP in 2009 but had graduated from the program by 2013 (34 percent, or 145 households) and those still benefiting from the program in 2013. This prompts the analysis to explore the heterogeneity of the results across PSNP graduation status, shown in the lower panel of table 2.

The associations between PSNP participation and improved math skills appears to be driven by graduated households. However, when considering FCS, both children in graduated households and current beneficiaries display significantly higher LTM and IL, compared to children in the control households. Children from nongraduated households also have significantly higher WM scores compared to children in the control group, but not in graduated households (and the point estimate is close to 0). Therefore, focusing only on domain-specific tests might lead to the incorrect conclusion that the PSNP only affects the cognitive ability of its graduates, but not its current beneficiaries. However, the results in table 2 provide suggestive evidence that the PSNP is able to affect domain-general FCS among both its graduates and current beneficiaries.^20^

The magnitudes of the FCS estimates presented in table 2 actually suggest that, if there are differences in the associations between graduated and nongraduated households, they may be larger among current beneficiaries. This may be attributed to the fact that many graduated households in the program's initial years were not yet food secure and had asset levels no different from those households who were still in the program. In fact, evidence suggests that many households were graduated out of the PSNP simply due to great political urgency to move fast with graduation and to show “success” in terms of both poverty reduction and value for money (Sabates-Wheeler et al. 2021). Table S.1.9 in the supplementary online appendix compares pre-program characteristics of the graduated and nongraduated households and suggests that, in accordance with this, graduated households were poorer before the introduction of the program. Given that contemporaneous food consumption and nutrition are associated with improved FCS (Benton and Parker 1998; Herzog et al. 2012), these differences in household wealth offer one potential explanation as to why the FCS associations might be larger among current beneficiaries.

### Heterogeneous by Gender

This paper next examines whether there are heterogeneous effects of the PSNP according to the gender of the child (table S.1.10 in the supplementary online appendix). The results suggest that the positive association between the PSNP and LTM is significantly larger among females, as the interaction term is negative and economically large (−0.295). Indeed, estimating the LTM regression separately by gender suggests that the results are entirely driven by a positive association among females.^21^ In contrast, there is no significant difference according to gender for the positive association between the PSNP and IL.

## 6. Potential Mechanisms

This section investigates two mechanisms that may explain the positive association between the PSNP and FCS (and more specifically LTM and IL). First, by increasing household income, the PSNP may positively impact cognition through an increase in children's food consumption and nutrition. Given that the PSNP explicitly targets food-insecure households, it is possible that the program plays a remediation role, affecting FCS through ameliorating past nutritional deficits among those who experienced deprivations early in life.

Second, the work requirement imposed by the program means that there may be changes in the time allocations of children, which could affect the formation of cognitive skills. For example, children may substitute in for adult labor in family businesses or household work, or they may work less at home as their parents are earning more stable incomes. When considering this mechanism, it is assumed that spending time working (paid or unpaid) or caring for others does not improve FCS (unlike, for example, time in school). The program may also affect time use of adults and time spent interacting with children, but unfortunately data are not available to test these hypotheses.

### Time-Use Reallocation

As discussed, the work requirement among adults imposed by the PSNP means that there may be changes in the time allocations of children that could affect their cognitive development. Favara, Porter, and Woldehanna, (2019) argue that the effect of the PSNP on cognitive test scores is at least partially related to time reallocation towards educational activities, as students in PSNP households spend significantly more time in schooling in 2013 than the comparison children.

To examine whether the positive associations between the PSNP and FCS may be driven by time reallocations, the PSNP treatment variable is interacted with information on time use before the introduction of the program (in 2006). Given the ages of the children before the program ($\sim$ five years),^22^ many children reported spending zero hours caring for others, performing domestic tasks (e.g., fetching water and firewood), or performing unpaid labor on the family farm/business.^23^ Therefore, a binary variable is generated for each time category (household responsibilities and unpaid labor) that takes the value of 1 if children reported *any* hours in the category, and 0 otherwise.^24^ For both LTM and IL, equation (2) is estimated, replacing $Mal{e}_{ihj}$ with each time-use binary variable. Table 3 reports the results.^25^

**Table 3.** Heterogenous Effects According to Pre-program Time Use (2006)

	Unpaid labor	Household responsibilities
	(1)	(2)
**Panel A. Long-term memory (LTM)**		
PSNP	0.195	0.283*
	(0.140)	(0.183)
PSNP * Reported any hours	−0.003	−0.148
	(0.224)	(0.182)
PSNP among those who reported any hours	0.192	0.135
	(0.232)	(0.148)
Observations	404	404
${R}^2$	0.249	0.252
**Panel B. Implicit learning (IL)**		
PSNP	0.043	0.132*
	(0.058)	(0.062)
PSNP * Reported any hours	0.239*	−0.001
	(0.146)	(0.067)
PSNP among those who reported any hours	0.282**	0.131*
	(0.117)	(0.061)
Observations	404	404
${R}^2$	0.747	0.745

*Source*: Authors’ analysis based on data from the Young Lives Study (YLS) in Ethiopia. Data on foundational cognitive skills (FCS) were obtained during the fourth YLS survey round in 2013. Data on pre-program time use are from the second YLS survey round in 2006.

*Note*: The table reports OLS estimates with standard errors (reported in parentheses) clustered at community level. Panel A reports the heterogeneous associations between participation in the Productive Safety Net Programme (PSNP) and LTM according to pre-program time-use; panel B reports the heterogeneous associations between participation in the PSNP and IL according to pre-program time-use. For both LTM and IL, equation (2) is estimated with each time-use binary variable. RACER outcomes are standardized using the mean and standard deviation of the control group. All estimations include community fixed effects, and control for the age and gender of the child, the child's main language, religion, and ethnicity, the socioeconomic status of the household, the household size, the household head's gender and education, whether the household owned their house and the land on which the house was built, the household's food expenditure, whether the household has access to a school, whether the household has an influential connection, the number of people the family can rely on for financial need, and whether the caregiver believes that a child should leave school for work if needed; *p-*values are calculated using wild bootstrap standard errors (clustered at the community level) derived from running 1,000 replications. “Reported any hours” takes the value of 1 if the participant reported any hours in the relevant time use category in 2006, and 0 otherwise. “PSNP among those who reported any hours” coefficient reflects the association between the PSNP and the FCS of children who reported spending at least some time in each time-use category in 2006. It is calculated as the linear combination of the PSNP and PSNP * Reported any hours regression coefficients. The sample size in this table is slightly reduced, as time-use information was not collected on children under five in 2006; 207 (33 percent) children in the sample were under five years old in 2006 and were not asked the time-use questions.

^***^Significant at 1 percent. ^**^Significant at 5 percent.^*^Significant at 10 percent.

In panel A (LTM), neither of the interaction terms is statistically different from 0, suggesting that the LTM result is unlikely to be related to time reallocations among PSNP beneficiary children. For IL, however, (panel B), the interaction term between the PSNP treatment variable and the dummy variable for any hours spent in unpaid labor is economically large and statistically significant (at the 10 percent level). The positive point estimate suggests that the association between the PSNP and IL is significantly larger among those who had unpaid labor responsibilities before the program. In fact, the PSNP coefficient is only statistically significant among those who reported any unpaid labor hours; among those with no unpaid labor hours in 2006, the PSNP coefficient is small and statistically insignificantly. Taken together, these findings imply that the IL result may, in part, be due to a reallocation of time away from unpaid labor responsibilities at home toward educational activities. In line with this interpretation, being a PSNP beneficiary is significantly associated with an increase in educational hours (in school and studying) between 2006 and 2013.^26^

### Remediation of Early-Life Nutritional Deficits

Using YLS data, Porter and Goyal (2016) find a positive medium-term association between PSNP participation and height-for-age z-scores for children aged 5–15, suggesting that the program positively impacted children's long-run nutritional outcomes.

As a first step, equation (1) is estimated using the body mass index (BMI) in 2013 as the outcome to assess whether, among the sample in this paper, being a PSNP beneficiary is associated with improved short-run nutritional status (table S.1.14 in the supplementary online appendix). Consistent with Porter and Goyal (2016), the results suggest that PSNP beneficiaries have significantly higher BMI scores; on average, being a PSNP beneficiary is associated with an increase in BMI of 0.24 kg/${{\mathrm{m}}}^2$.^27^ Given that the beneficiaries tend to be undernourished, this is a positive gain.

To test the possible remediation role of the program, the PSNP treatment is interacted with information on whether the participants were stunted before their households started receiving the program (in 2006).^28^ Stunting is a well-established measure of individual health status, especially among children, which typically reflects the persistent, cumulative effects of inadequate nutrition (e.g., Hoddinott et al. 2013). Children are considered stunted if their heights are more than two standard deviations below the World Health Organization medians for a well-nourished population (WHO 2006). Table 4 reports the results.

**Table 4.** Heterogenous Effects According to Pre-program Nutritional Status

	(1)	(2)
	LTM	IL
**Panel A. Pre-program stunting status (2006)**		
PSNP	0.038	0.068
	(0.093)	(0.062)
Stunted (2006)	−0.426**	−0.063
	(0.164)	(0.074)
PSNP*Stunted (2006)	0.441**	0.141
	(0.161)	(0.090)
PSNP among stunted (2006)	0.479***	0.209***
	(0.125)	(0.068)
Observations	608	608
**Panel B. Early-life rainfall shocks**		
*In-utero shock*		
PSNP	0.124*	0.125**
	(0.067)	(0.057)
In-utero shock	−0.110	0.020
	(0.361)	(0.062)
PSNP*in-utero shock	0.281	−0.088
	(0.184)	(0.069)
*Year 1 shock*		
PSNP	0.047	0.129**
	(0.057)	(0.052)
Year 1 shock	−0.249	0.088
	(0.152)	(0.055)
PSNP*Year 1 shock	0.414***	−0.067
	(0.101)	(0.113)
*Year 2 shock*		
PSNP	0.122	0.173**
	(0.109)	(0.103)
Year 2 shock	−0.534	−0.037
	(0.668)	(0.123)
PSNP * Year 2 shock	0.081	−0.105
	(0.213)	(0.145)
Observations	512	512

*Source*: Authors’ analysis based on data from the Young Lives Study (YLS) in Ethiopia and rainfall data from the University of Delaware (Matsuura and Willmott 2018). Data on foundational cognitive skills (FCS) were obtained during the fourth YLS survey round in 2013.

*Notes*: The table reports OLS estimates with standard errors (reported in parentheses) clustered at community level. LTM = long-term memory, IL = implicit learning. Panel A reports the heterogeneous associations between participation in the Productive Safety Net Programme (PSNP) and FCS according to pre-program stunting status; panel B reports the heterogeneous associations between participation in the PSNP and FCS according to pre-program exposure to rainfall shocks. RACER outcomes are standardized using the means and standard deviations of the control group. All estimations include community fixed effects, and control for the age and gender of the child, the child's main language, religion and ethnicity, the socio-economic status of the household, the household size, the household head's gender and education, whether the household owned their house and the land on which the house was built, the household's food expenditure, whether the household has access to a school, whether the household has an influential connection, the number of people the family can rely on for financial need, and whether the caregiver believes that a child should leave school for work if needed; *p-*values are calculated using wild bootstrap standard errors (clustered at the community level) derived from running 1,000 replications. “Stunted (2006)” takes the value of 1 if the participant was stunted in 2006, and 0 otherwise. “PSNP among stunted (2006)” coefficient reflects the effect of the PSNP on FCS of children who were stunted in 2006. It is calculated as the linear combination of the PSNP and PSNP*Stunted (2006) coefficients. Rainfall analysis is only performed on children who have complete community GPS information. For each community, a rainfall shock is defined as any monthly Standardized Precipitation Index deviation of at least two standard deviations above or below the historical monthly average of the same community.

***Significant at 1 percent. **Significant at 5 percent. *Significant at 10 percent.

The results in panel A suggest that the association between the PSNP and LTM is significantly larger for those who were stunted before receiving the program. For both tasks, the PSNP coefficient is positive and significant among those who were stunted in 2006; in contrast, for both FCS tasks, the PSNP coefficient among those who were not stunted in 2006 is small and statistically insignificant. This provides suggestive evidence that the observed effect of the PSNP on FCS may, in part, be due to the program's success in remediating past nutritional deficits (particularly for LTM).

### Remediation of Early-Life Rainfall Shocks

The above results provide suggestive evidence that the positive association between the PSNP on LTM may be due to improvements in nutrition. This raises the question of whether part of the program's success may be due to the remediation of negative effects due to early-life rainfall shocks, which may have affected nutritional and other investments in children. With 80 percent of the population living in rural areas and relying on rain-fed agriculture, Ethiopia is highly vulnerable to extreme climate condition (UN DESA 2019). In recent decades, the country has been exposed to multiple, severe droughts—with adverse short- and long-term consequences (Webb and Braun 1994; Dercon 2004; Porter 2012). In particular, a growing body of international evidence finds that early-life rainfall shocks negatively impact children's nutritional (e.g., Dercon and Porter 2014; Yamano, Alderman, and Christiaensen 2005; Dimitrova and Muttarak 2020) and educational (e.g., Adhvaryu et al. 2023; Duque, Rosales-Rueda, and Sanchez 2019) outcomes, suggesting that the PSNP have been able to affect FCS through partially offsetting the negative nutritional, as well as possibly other, effects of rainfall shocks.

To analyze whether part of the PSNP's positive effects on FCS may be due to the remediation of early-life rainfall shocks, the YLS-data is combined with gridded data on monthly precipitation to generate monthly community-level rainfall estimates. Rainfall data are obtained from the University of Delaware (accessed at https://psl.noaa.gov), a commonly used climate dataset in the literature (e.g., Rocha and Soares 2015; Shah and Steinberg 2017), which contains high-spatial-resolution (0.5°) gridded estimates of monthly total precipitation across land surfaces between 1900 and 2014 (Matsuura and Willmott 2018). For each YLS community, the survey collected GPS coordinates using, as a reference point, the center of the community, either identified as the center of the main square or, in the absence of it, of another point of interest (e.g., city hall, school, post office, church). Using this information, the GPS locations of the YLS communities were matched to the rainfall data grid points, using the main square in each community as the reference point. For each community, monthly rainfall precipitation was calculated as an inverse-distance-weighted average of the monthly rainfall registered at the four closest grid points to that community.

To identify exogenous rainfall shocks and their intensity, for each community, a Standardised Precipitation Index (SPI) was constructed. The SPI was first proposed by McKee, Doesken, and Kleist (1993) and is recommended by the World Meteorological Organisation for the characterization of meteorological droughts (Wu et al. 2007). The SPI derives a value for a month's rainfall in terms of standard deviations from the long-term mean of the transformed standardized normal distribution for that month-of-year and community specifically. Deviations from the mean are more relevant than absolute rainfall because individual and communities typically adapt to local conditions on average (e.g., with regard to composition of agricultural products). The SPI is preferred to using raw precipitation data as, unlike a deviation from the simple long-term average, the non-negative and positively skewed nature of rainfall is accounted for prior to normalization. Another advantage of this measure is that it requires only precipitation to calculate and is computationally simple, unlike other measures such as the Palmer Drought Index (Lloyd-Hughes and Saunders 2002). To calculate the SPI, precipitation data are fitted to a gamma distribution, and then transformed to a standard normal distribution with a mean value of 0 (McKee. Doesken, and Kleist 1993; Agnew 2000). This is conducted for each month of the year at each community separately, providing a month-community specific measure of rainfall anomalies relative to long-run conditions.

Following the definition of an “extreme” weather condition (“extremely wet” or “extremely dry”) in McKee, Doesken, and Kleist (1993), for each community, a rainfall shock is defined as any monthly SPI deviation of at least two standard deviations above or below the historical monthly average of the same community. For each YLS respondent, information about the date and place of birth is used to identify whether children experienced at least one rainfall shock during the gestation (in-utero, pre-natal) period and/or during the early childhood period (the first and second years of life after birth).^29^ For LTM and IL, equation (2) is then estimated three times, replacing $Mal{e}_{ihj}$ with a binary variable that takes the value of 1 if children experienced at least one rainfall shock during the in-utero period, the first year of life, or the second year of life, respectively. This tests whether the association of the PSNP with improved LTM and IL differs according to the experience of adverse exogenous shocks during early childhood.^30^ These results are shown in the lower panel of table 4.

For LTM, the interaction term is positive and statistically significant in the 'Year 1 shock' panel, suggesting that the association between PSNP and LTM is significantly larger for those who experienced at least one rainfall shock during their first year of life. In contrast, none of the interaction coefficients are statistically insignificant for IL.^31^

One interpretation of these results is that, while a lower level of LTM may be observed among those exposed to early-life rainfall shocks, this negative effect is partially offset within the group of PSNP-recipients. This, combined with the findings in panel A, provides evidence that the positive association between the PSNP and LTM may, at least in part, be due to the program's success in remediating early-life deficits in nutritional and other investments in children, caused by rainfall shocks. Indeed, early-life rainfall shocks likely represent one important determinant of malnutrition, as 59 percent of children who were stunted at age 5 experienced at least one rainfall shock during their first 1,000 days after conception.^32^

## 7. Conclusion

This paper finds significant, positive associations of a large public works program, the Ethiopian PSNP, with foundational cognitive skills of young adolescents who grew up in poverty, but received transfers from the program during their childhood. These associations are most robust for long-term memory and implicit learning, and positive, but weaker for working memory. This paper also finds suggestive evidence that, in part, the association with implicit learning may be operating through the income effect of the program allowing time reallocation away from unpaid labor hours, while the association with long-term memory may be due to the program's success in remediating past nutritional deficits caused by early-life rainfall shocks.

There are a number of key limitations in the analysis, related to the fact that this paper only observed foundational cognitive skills in one time period. First, it is not able to utilize longitudinal data techniques (such as individual fixed effects). Second, it is not able to analyze dynamics over time, to assess whether the observed associations persist into adolescence and early adulthood. Last, given their high vulnerability, PSNP beneficiaries might be enrolled in other programs at the same time (e.g., the Community-Based Health Insurance and the Health Extension Programme). Given the data, this paper is unable to definitively rule out that the observed associations are driven by the PSNP alone.^33^

In spite of the limitations described above, the link between enrolment in the PSNP and foundational cognitive skills is relevant for the future design and targeting of the PSNP, and other similar public works programs. Taken together, the findings in this paper suggest that Public Works Programs can have positive effects on child foundational cognitive skills, and in particular, that policy interventions may be able to mitigate the effects of early poverty on cognitive skills formation, and be able to improve children's potential future outcomes. Recent changes to make the PSNP more nutrition-sensitive, combined with targets for improved delivery, may increase the effectiveness of the program for children in future (Roelen et al. 2017).

## Conflicts of Interest

The authors have no conflicts of interest to report.

## Data Availability Statement

The Young Lives household survey data used in this article are publicly archived and available to download from the UK Data Archive, at https://ukdataservice.ac.uk. The foundational cognitive skills data are set to be publicly archived by March 2025. Before then, data will be shared on request to the author with permission from Young Lives. The rainfall data used in this article are from the University of Delaware, and can be accessed at https://psl.noaa.gov. Matched rainfall data to Young Lives respondents are set to be publicly archived by March 2024. Before then, data will be shared on request to the author with permission from Young Lives.

## Supplementary Material

lhad035_Supplementary_Online_Appendix

## Footnotes

1 FCS are a set of cognitive abilities that are considered domain-general, rather than skills such as reading, arithmetic, or linguistics, which are domain-specific to certain types of knowledge (Behrman et al. 2022). FCS capture fluid intelligence skills (such as the ability to reason abstractly and solve novel problems), while domain-specific cognitive achievement tests assess crystallized intelligence skills (which involve knowledge that comes from prior learning and past experiences).

2 And it should be noted that none of the survey participants owned a tablet, so all were equally unfamiliar with the exercise.

3 Initially, a *woreda* was considered chronically food insecure if (a) it was in one of eight selected regions (Tigray, Amhara, Oromiya, SNNP, Afar, Somali, rural Harari and Dire Dawa), and (b) had been a recipient of food aid for a significant period, generally for at least three years (Sharp, Brown, and Teshome 2006).

4 The PSNP operates a “scalable” safety net, scaling-up assistance to an average of 3.8 million people annually, with the eventual ambition that it will scale-up to a national rural program (Ministry of Agriculture 2020). Consequently, in 2013, there were food-insecure households that did not have access to the PSNP simply because the program had not been scaled-up to their *woredas* yet.

5 To achieve such low levels of attrition, the study followed families that moved within the country (Sánchez and Escobal 2020).

6 This information was investigated to see whether it could be used to establish more nuanced treatment variables, but it was too incomplete to be of use.

7 In the sample in this paper, however, it is unlikely that familiarity with electronics materially accounts for differences in RACER scores, given that no households owned a computer or laptop in 2013.

8 In comparison, the Peabody Picture Vocabulary Test (PPVT), which measures receptive vocabulary, was successfully administered to 88 percent of the children.

9 An investigation was conducted to assess the feasibility of treating public-works employment and direct-support households separately. However, the sample size of households receiving direct support was too small to examine on its own.

10 Sample sizes change slightly for different outcomes, due to missing information and sample restrictions. Table S.1.2 in the supplementary online appendix provides details on the sample sizes for each outcome.

11
Gilligan, Hoddinott, and Taffesse (2009) highlighted that the PSNP transfers were delayed during the first year of implementation (2005/6), and impact was not experienced in the YLS Round 2 (Woldehanna 2010), motivating this study to use 2006 (YLS Round 2) as the pre-program comparison year.

12 The YLS wealth index takes values between 0 and 1, such that a larger value reflects a wealthier household. It is the simple average of a housing-quality index, an access-to-services index, and a consumer-durables index (Briones 2017).

13 This variable is based on a question along these lines: “Imagine that a family in the village has a 12 year old son/daughter who is attending school full-time. The family badly needs to increase the household income, and one option is to send the son/daughter to work but the son/daughter wants to stay in school. What should the family do?” A full descriptive statistics balance table, with all variables shown, appears in table S.1.3 in the supplementary online appendix.

14 It is also possible that FCS are influenced by the fieldworker who administered the FCS tests. For example, the tests may be affected by the fieldworker's expression skills, as the tests are administered based on demonstration. The main results in this paper are robust to including fieldworker fixed effects (Table S.1.4 in the supplementary online appendix).

15 Not all children successfully completed all RACER tasks. Hence, the number of observations varies slightly across RACER measures.

16 As a robustness check for the LTM and IL results, this paper applies the methodology suggested by Oster (2019) and compares the coefficient movements and changes in the R-squared when the full set of controls are included to when only game (RACER) controls are included. Overall, the results (shown in table S.1.5 in the supplementary online appendix) reinforce the likelihood that, if the results in table 2 are biased, they are likely to understate the magnitude of the PSNP's association with LTM and IL.

17 Some studies that aim to estimate a causal impact of the PSNP use propensity-score matching (PSM) to construct an appropriate counterfactual rather than constructing a restricted sample (e.g., Gilligan, Hoddinott, and Taffesse, 2009; Hoddinott, Gilligan and Taffesse 2010; Woldehanna 2010). The robustness of the findings in table 2 are assessed using PSM rather than the restricted control sample (results in table S.1.6, table S.1.7, and fig. S.1.2 in the supplementary online appendix). Using PSM does little to alter the interpretation of the results.

18 Although the PSNP enrolment has targeting guidelines, several studies (e.g., Coll-Black et al. 2011) have shown that a considerable proportion of PSNP beneficiaries were nonpoor and suggested frequent retargeting. To assess the robustness to potential estimation biases arising from targeting issues, the results are re-estimated excluding the top 20 percent of participants based on the 2006 wealth index. The results are robust to this sample restriction (table S.1.8 in the supplementary online appendix).

19 Both the PPVT and mathematics scores are constructed using Item Response Theory (IRT) models, which are commonly used in international assessments such as PISA and TIMSS (see Leon (2020) for more details). These results come from an estimation of equation (1), excluding the weekday and the time of the day when the FCS tasks were administered.

20 The math coefficient among current beneficiaries is positive and nearly statistically significant at the 10 percent level (*p-*value = 0.16). Previous evidence (e.g., López et al. 2022) finds that FCS are a key determinant of math test scores, suggesting that the PSNP might lead also to an improvement of math among the current beneficiaries, but that this effect might take longer to be revealed than FCS.

21 Separate regressions by gender for the association between the PSNP and LTM are shown in table S.1.11 in the supplementary online appendix.

22 In 2006, index children were, on average, 5.2 years old.

23 Information on pre-program educational activities (in school or studying) and paid work was also considered, but too few children engaged in these categories in 2006 for meaningful analysis.

24 The full distributions for number of reported hours in each category are shown in table S.1.12 in the supplementary online appendix.

25 The adult work requirement is only applicable to households in the public-works aspect of the PSNP, not to those that receive direct support. In 2013, 12 percent of children receiving the PSNP reported being in households receiving direct support. To ensure that the results in table 3 are not unduly affected by children receiving direct support, the analysis was re-estimated on the sample of children in public-works households only. Overall, the interpretation of the findings is robust to this sample limitation (results in table S.1.13 in the supplementary online appendix).

26 Results shown in table S.1.17 in the supplementary online appendix.

27 An earlier impact evaluation (Berhane et al. 2017) does not find any impact of the PSNP on child nutritional status. However, there are a number of important differences in the data collection. Firstly, anthropometric outcomes were not collected in 2006, meaning that the Berhane et al. (2017) evaluation had no baseline/pre-program data. Secondly, anthropometric measures were collected in 2008–2012 for all children living in respondent households—but only in the age range of six months to five years. Given these differences, the results in this paper on nutrition likely represent the longer-term associations of the PSNP from age 5 through age 12. The Berhane et al. (2017) results can be interpreted that children who have higher birth order in the PSNP households do not appear to have better nutrition over time; this may be conflating the PSNP effect with a birth-order effect, whereby there is often an advantage to earlier-born children.

28 This is estimated using equation (2), replacing $Mal{e}_{ihj}$ with an indicator that takes the value of 1 if the child was stunted in 2006, and 0 otherwise. In addition to the regular controls, the regression also controls for pre-program (2006) BMI.

29 Eight children moved/migrated from communities between conception and Round 1 (children ∼ 6–18 months old), or between Round 1 and Round 2 (children ∼ 4.5–5.5 years old). Rather than dropping these children, they have been kept in the analysis and assigned the rainfall patterns of their original communities. However, 96 children are excluded, as they did not have complete community GPS information.

30 In contrast with early-life undernutrition, the experience of a rainfall shock is likely exogenous to the household. Consequently, this analysis provides a more robust methodology to assess the remediation role of the PSNP on FCS in the presence of early-life deprivations.

31 Previous research shows that results can be sensitive to how rainfall shocks are defined (Sohnesen 2020). Therefore, in table S.1.15 in the supplementary online appendix, the findings are shown to be robust to using 2.5 SPI as a cut-off for defining a rainfall shock.

32 The Pearson correlation coefficient between stunting status at age 5 and rainfall shocks during the first 1,000 days is 0.144, significant at the 1 percent level.

33 As one robustness check, however, the main results are re-estimated controlling for whether, in 2013, the household reporting receiving the Health Extension Programme, Emergency Aid, or any other social protection program. The main results are robust to this alternative specification (table S.1.16 in the supplementary online appendix).

## Author Biography

Richard Freund (corresponding author) is a PhD candidate at the University of Cape Town School of Economics, Cape Town, South Africa, 7701, and is a Research Affiliate at the Oxford Department of International Development; his email address is FRNRIC009@myuct.ac.za. Marta Favara is a Senior Research Officer at the Oxford Department of International Development, University of Oxford, Oxford, UK, OX1 3TB, and is the Director of Research at Young Lives; her email address is marta.favara@qeh.ox.ac.uk. Catherine Porter is the Director of Young Lives and Professor in Development Economics at the Lancaster University Management School, Lancaster, UK, LA1 4YX; her email address is catherine.porter@qeh.ox.ac.uk. Jere Behrman is the William R. Kenan, Jr. Professor of Economics at the University of Pennsylvania School of Arts and Science, Philadelphia, USA, PA 19104; his email address is jbehrman@econ.upenn.edu. The authors have nothing to disclose. This study was funded by the Eunice Kennedy Shriver National Institute of Child Health and Human Development [NICHD R21 HD097576]. Thanks also to the UK's Foreign, Commonwealth and Development Office (FCDO) for funding Young Lives at Work and enabling this research (grant number GB-GOV-1-301108). Freund also acknowledges funding from the Oppenheimer Memorial Trust and the University of Cape Town. Funders had no role in the design, interpretation, or writing-up of the study, or in the decision to submit the study for consideration for publication. The Institutional Review Board of the University of Pennsylvania provided ethical approval for the project (protocol #834313). Special thanks to Margaret Sheridan, Douglas Scott, Alan Sánchez, Annina Hittmeyer, Nicolás Pazos, Jennifer Lopez, Santiago Cueto, Tassew Woldehanna, and Alula Pankhurst for useful comments and feedback. A supplementary online appendix for this article can be found at *The World Bank Economic Review* website.
