# Supplementary material for: Social Protection and Foundational Cognitive Skills during Adolescence: Evidence from a Large Public Works Program
Source: World Bank Econ Rev. 2023 Nov 13;38(2):296–318. doi: 10.1093/wber/lhad035 (PMC11057409; doi:10.1093/wber/lhad035)
Supplement: lhad035_Supplementary_Online_Appendix [file lhad035_supplementary_online_appendix.pdf]

Supplementary Online Appendix

Social Protection and Foundational Cognitive Skills during Adolescence: Evidence from a Large Public Works Program

Richard Freund, Marta Favara, Catherine Porter, and Jere Behrman

S1: Additional References, Figure, and Tables

Figure S1.1. RACER in Practice.

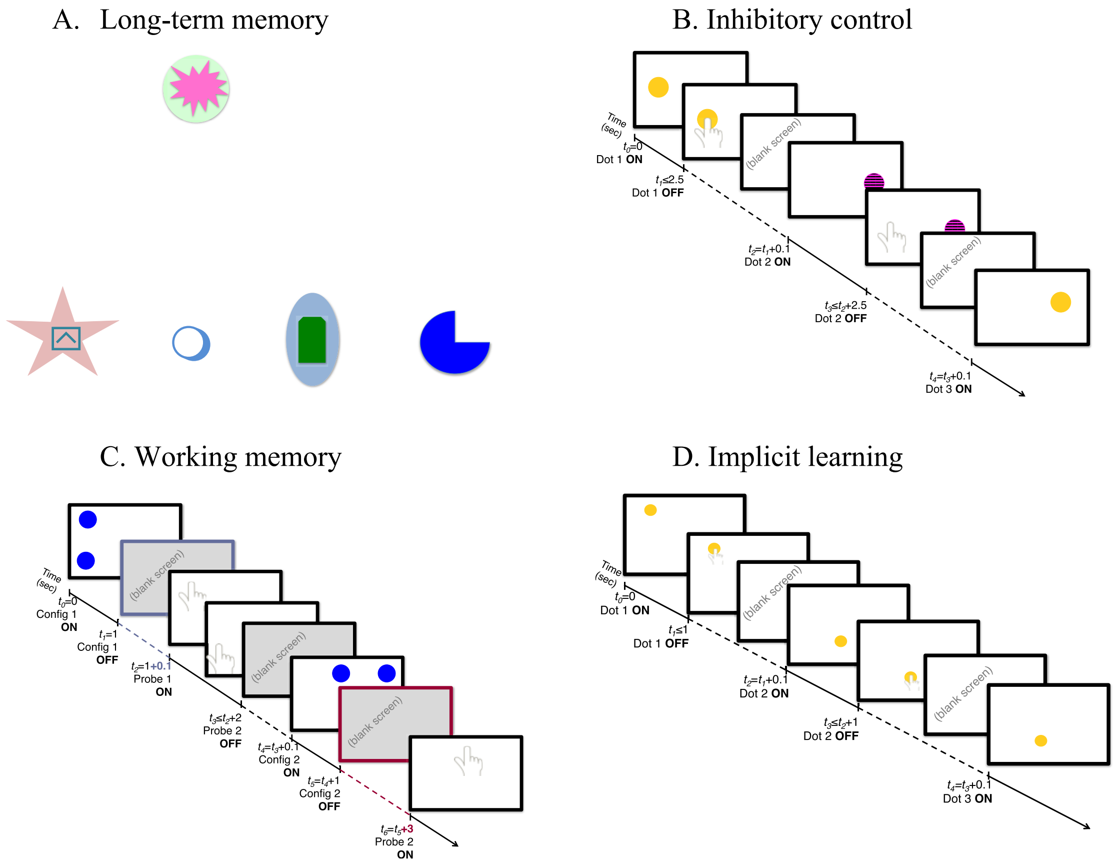

Source: Figure adapted from Behrman et al. (2022).

Figure S.1.2. Propensity Score Common Support.

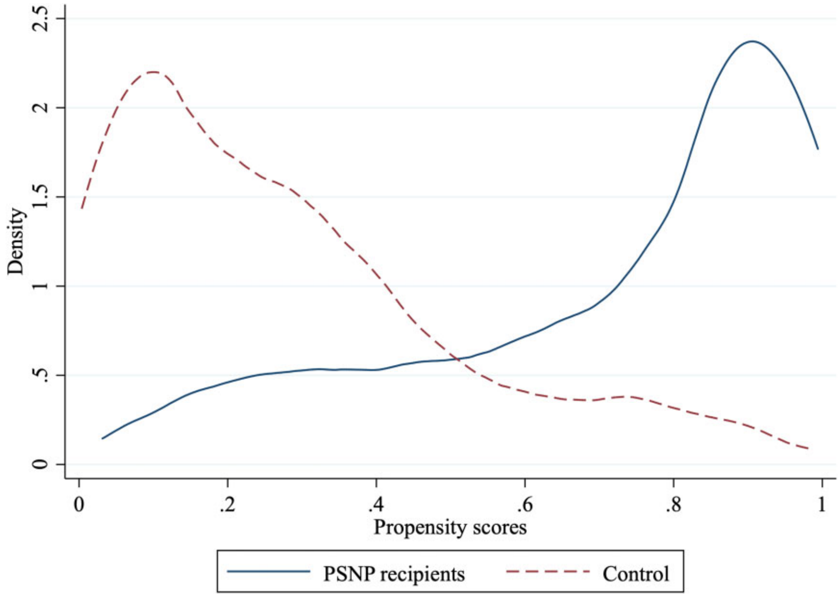

Source: Authors' analysis based on data from the Young Lives Study (YLS) in Ethiopia.
Note: Propensity scores are estimated using a logistic regression and information on pre-PSNP (2006) wealth index score (Briones 2017), pre-primary education attendance, community of residence, and negative economic shocks (crop failure, death of livestock, and illness or death of household member).

Table S.1.1. Comparison of Non-PSNP Households and PSNP Households: Pre-Program Balance

	Non-PSNP households	PSNP households
Male	0.53	0.53
Household size	6.29	6.24
Head of house schooling grade	2.24	1.27***
Male head of house	0.93	0.81***
Lowest wealth income tercile	0.46	0.50
Middle wealth income tercile	0.40	0.43
Top wealth income tercile	0.14	0.08***
Owns livestock	0.90	0.94
Owns land of house	0.96	0.91***
Monthly per capita nonfood expenditure	32.69	21.71***
Access to electricity	0.18	0.11***
Observations	721	479

Source: Data comes from the Young Lives Study (YLS) in Ethiopia.
Note: All variables are measured in YLS Round 2 (2006). Wealth terciles are based on the Young Lives wealth index (Briones 2017). Asterisks reflect *p*-values for a *t*-test for differences in means between non-PSNP households and PSNP households.
***Significant at 1 percent. **Significant at 5 percent. *Significant at 10 percent.

Table S.1.2. Sample Sizes for Outcome Variables

Outcome	Restricted control	PSNP	Still receiving PSNP (2013)	Graduated from PSNP	Regression sample size
Long-term memory (2013)	186	422	277	145	608
Inhibitory control (2013)	187	422	277	145	609
Working memory (2013)	186	422	277	145	608
Implicit learning (2013)	187	421	277	144	608
PPVT (2013)	139	388	251	137	527
Maths (2013)	187	422	277	145	609
Unpaid labor (2006)	133	272	182	90	404
Household responsibilities (2006)	133	272	182	90	404
BMI (2013)	187	422	277	145	609
Stunted (2006)	187	422	277	145	608
Rainfall shock in-utero	166	347	213	134	512
Rainfall shock in year one	166	347	213	134	512
Rainfall shock in year two	166	347	213	134	512
Sample size	187	422	277	145	

Source: Survey data comes from the Young Lives Study (YLS) in Ethiopia, and rainfall data comes from the University of Delaware (Matsuura and Willmott 2018). Data on foundational cognitive skills (FCS) were obtained during the fourth YLS survey round in 2013.

Note: The lower response rate for PPVT is due to the fact that the test was not administered if the child's language was not Amharic, Oromifa, or Tigrinya. The reduced sample size for Unpaid labor, and Household responsibilities is because time-use information was not collected on children who were under five in 2006. The lower sample size for the rainfall shock variables is because they are only defined for children with complete GPS information who did not move communities between conception and 2006.

Table S.1.3. Descriptive Statistics (Full)

Variable	PSNP	Control
**Panel A. Background characteristics (2006)**		
Child's age (in years)	4.65	4.73
	(0.48)	(0.46)
Male child	0.54	0.55
	(0.50)	(0.50)
Household size	6.32	6.39
	(1.84)	(1.88)
Head of house schooling grade	1.33	2.09***
	(2.16)	(2.79)
Male head of house	0.84	0.96***
	(0.37)	(0.19)
Monthly food expenditure (birr)	61.28	64.14
	(44.03)	(42.42)
Amhara ethnicity	0.19	0.42***
	(0.40)	(0.50)
Oromo ethnicity	0.17	0.09**
	(0.37)	(0.29)
Tigrayan ethnicity	0.50	0.13***
	(0.50)	(0.34)
Wealth index score	0.19	0.19
	(0.11)	(0.11)
Owns livestock	0.91	0.96
	(0.29)	(0.19)
Owns land of house	0.91	0.97**
	(0.28)	(0.16)
Owns house	0.93	0.95
	(0.25)	(0.23)
Access to electricity	0.11	0.07
	(0.31)	(0.26)
Parent has regular salaried employment	0.02	0.04
	(0.14)	(0.19)
Parent works in agriculture	0.18	0.20
	(0.38)	(0.40)
Has access to schools	0.98	0.98
	(0.13)	(0.14)
Household has influential relative/friend	0.61	0.59
	(0.49)	(0.49)
Number of people can rely on for financial need	2.01	2.09
	(1.50)	(1.49)
Believes child should leave school to work if needed	0.12	0.12
	(0.33)	(0.32)
**Panel B. Intermediate outcomes (2006)**		
Body Mass Index	14.53	14.57
	(1.46)	(1.59)
Child is stunted	0.35	0.37
	(0.48)	(0.49)
Exposed to early life rainfall shock (first 1,000 days)	0.37	0.80***
	(0.48)	(0.40)
Engaged in unpaid labor	0.31	0.38
	(0.46)	(0.49)
Engaged in household responsibilities	0.50	0.48
	(0.50)	(0.50)

Table S.1.3. Continued

Variable	PSNP	Control
**Panel C. RACER outcomes (2013)**		
Long-term memory	0.01	−0.02
	(1.12)	(0.99)
Inhibitory control	−0.19	0.01***
	(0.69)	(0.72)
Working memory	0.03	0.02
	(1.01)	(0.97)
Implicit learning	−0.02	0.00
	(1.00)	(1.00)
Long-term memory (baseline)	−0.08	−0.02
	(0.99)	(1.00)
Inhibitory control (baseline)	−0.10	0.02*
	(0.70)	(0.68)
Working memory (baseline)	−0.00	−0.00
	(1.04)	(1.00)
Implicit learning (baseline)	−0.03	0.01
	(0.97)	(1.02)
Number of children (full sample)	422	187

Source: Data comes from the Young Lives Study (YLS) in Ethiopia. Data on foundational cognitive skills (FCS) were obtained during the fourth YLS survey round in 2013.

Note: Panel A reports the summary statistics of the background (pre-program) characteristics of the survey respondents. Wealth terciles are based on the Young Lives wealth index (Briones 2017). Panel B reports the summary statistics of the FCS variables. FCS scores are standardized, according to the distributions for the non-PSNP children. columns (1) and (2) report the mean and standard deviation (in parentheses). Body Mass Index is calculated by dividing weight in kilograms by the square of height in meters. Children are considered stunted if their heights are more than two standard deviations below the World Health Organization medians for a well-nourished population (WHO Multicentre Growth Reference Study Group 2006). "Exposed to early life rainfall shock" takes the value of 1 if the participant experienced at least one month in the in-utero period, or first or second years of life, where the Standardized Precipitation Index deviated at least 2 standard deviations above or below the historical monthly average of the same community. "Engaged in unpaid labor" takes the value of 1 if children report spending in tasks on the family farm or business, and 0 otherwise. "Engaged in household responsibilities" takes the value of 1 if children report spending time caring for others or in domestic tasks (fetching water, firewood, cleaning, cooking, washing, shopping, etc.), and 0 otherwise. Asterisks reflect p-values for t-tests for differences in means between children in PSNP households and control households.

***Significant at 1 percent. **Significant at 5 percent. *Significant at 10 percent.

Table S.1.4. Robustness of Associations between the PSNP and FCS Using Fieldworker Fixed Effects

	LTM	IC	WM	IL
PSNP	0.183**	−0.042	0.026	0.137***
	(0.080)	(0.052)	(0.052)	(0.051)
Fieldworker fixed effects	Yes	Yes	Yes	Yes
Controls	Yes	Yes	Yes	Yes
Observations	608	609	608	608
R^2	0.309	0.452	0.344	0.754

Source: Authors' analysis based on data from the Young Lives Study (YLS) in Ethiopia. Data on foundational cognitive skills (FCS) were obtained during the fourth YLS survey round in 2013.

Note: The table reports OLS estimates from equation (1), controlling for the fieldworker who administered the RACER tests. Robust standard errors (reported in parentheses) are clustered at community level. LTM = long-term memory, IC = inhibitory control, WM = working memory, IL = implicit learning. RACER outcomes are standardized using the means and standard deviations of the control group. Each coefficient comes from a different estimation of equation (1) for each FCS outcome. All estimations include community fixed effects, and control for the age and gender of the child, the child's main language, religion, and ethnicity, the socioeconomic status of the household, the household size, the household head's gender and education, whether the household owned their house and the land on which the house was built, the household's food expenditures, whether the household has access to a school, whether the household has an influential connection, the number of people the family can rely on for financial need, and whether the caregiver believes that a child should leave school for work if needed; p-values are calculated using wild bootstrap standard errors (clustered at the community level) derived from running 1,000 replications.

***Significant at 1 percent. **Significant at 5 percent. *Significant at 10 percent.

Table S.1.5. Robustness of Results to Oster (2019) Sensitivity Analysis

Treatment variable, outcome variable	OLS estimate (Std. error), [R2˙] (1)	Controlled estimate $\tilde{\beta}$ (Std. error), $[\widetilde{R^2}]$ (2)	Identified set, $[\tilde{\beta}, \beta^*(1.3\widetilde{R^2})](3)$ (3)	Identified set excludes zero? (4)
PSNP, LTM	0.082 (0.081)[0.151]	0.211** (0.078)[0.245]	[0.211, 0.419]	Yes
PSNP, IL	0.010 (0.051)[0.720]	0.123** (0.051)[0.746]	[0.122, 4.151]	Yes

Source: Authors' analysis based on data from the Young Lives Study (YLS) in Ethiopia. Data on foundational cognitive skills (FCS) were obtained during the fourth YLS survey round in 2013.

Note: The OLS regressions (column 1) have only RACER baseline and game controls. The controlled effect is estimated using equation (1), including community fixed effects, and controlling for baseline measures, game controls, age, gender, main language, religion and ethnicity, wealth index score in 2006 (before the PSNP), household size, the household head's gender and education, whether the household owned their house and the land on which the house was built, household food expenditure, whether the household had access to a school, whether the household has an influential connection, the number of people the family can rely on for financial need, and whether the caregiver believes that a child should leave school for work if needed. LTM = long-term memory, IL = implicit learning. RACER outcomes are standardized using the means and standard deviations of the control group. The identified set in column (3) is bounded below by $\tilde{\beta}$ and above by β^* calculated based on $R_{max} = 1.3\widetilde{R^2}$ and $\delta = 1$ (Oster 2019). β^* was calculated using the Stata command psacalc provided by Oster (2019). Robust standard errors are clustered at the community level.

***Significant at 1 percent. **Significant at 5 percent. *Significant at 10 percent.

Table S.1.6. Robustness of Associations between the PSNP and FCS Using Propensity-Score Matching

	LTM	IC	WM	IL
PSNP (δ)	0.138*	0.021	0.038	0.136**
	(0.071)	(0.075)	(0.86)	(0.050)
Controls	Yes	Yes	Yes	Yes
Observations	803	804	803	803
R^2	0.401	0.492	0.393	0.783

Source: Authors' analysis based on data from the Young Lives Study (YLS) in Ethiopia. Data on foundational cognitive skills (FCS) were obtained during the fourth YLS survey round in 2013.

Note: The table reports OLS estimates from equation (1) weighted using inverse probability weights generated from propensity scores. Standard errors (reported in parentheses) are clustered at community level. Propensity scores are estimated using information on pre-PSNP (2006) wealth index score, pre-primary education attendance, community of residence, and negative economic shocks (crop failure, death of livestock, and illness or death of household member). RACER outcomes are standardized using the means and standard deviations of all children in non-PSNP households. Each coefficient estimate comes from a different estimation of equation (1), for each FCS outcome. All estimations include community fixed effects, and control for the age and gender of the child, the child's main language, religion, and ethnicity, the socio-economic status of the household, the household size, the household head's gender and education, whether the household owned their house and the land on which the house was built, the household's food expenditure, whether the household has access to a school, whether the household has an influential connection, the number of people the family can rely on for financial need, and whether the caregiver believes that a child should leave school for work if needed; p-values are calculated using wild bootstrap standard errors (clustered at the community level) derived from running 1,000 replications.

***Significant at 1 percent. **Significant at 5 percent. *Significant at 10 percent.

Table S.1.7. Logistic Model for Probability of Participating in the PSNP

	Dependent variable: PSNP participation
Number of males aged 0–5	−0.212
	(0.181)
Number of males aged 6–12	−0.371***
	(0.133)
Number of males aged 13–17	−0.050
	(0.167)
Number of males aged 18–60	−0.495***
	(0.175)
Number of males aged 61+	−0.379
	(0.468)
Number of females aged 0–5	−0.027
	(0.182)
Number of females aged 6–12	−0.078
	(0.125)
Number of females aged 13–17	0.082
	(0.178)
Number of females aged 18–60	−0.102
	(0.212)
Number of females aged 61+	0.455
	(0.483)
Shock (crops failed) 2002–2006	0.396*
	(0.204)
Shock (death of livestock) 2002–2006	−0.240
	(0.192)
Shock (death of father) 2002–2006	−0.044
	(0.714)
Shock (death of mother) 2002–2006	0.256
	(0.749)
Shock (death of other household member) 2002–2006	0.258
	(0.453)
Shock (illness of father) 2002–2006	0.248
	(0.271)
Shock (illness of mother) 2002–2006	−0.332
	(0.269)
Shock (illness of other household member) 2002–2006	0.063
	(0.250)
Household wealth index score (2006)	−7.241***
	(1.049)
Attended pre-primary education	0.133
	(0.595)
Community dummies (2006)	Yes
Pseudo R^2	0.378
Log likelihood	−386.130
Observations	895

Source: Authors' analysis based on data from the Young Lives Study (YLS) in Ethiopia.

Note: Propensity scores are estimated using a logistic regression.

***Significant at 1 percent. **Significant at 5 percent. *Significant at 10 percent.

Table S.1.8. Robustness of Associations between the PSNP and FCS Dropping Top 20 Percent of 2006 Wealth Index Distribution

	LTM	IC	WM	IL
PSNP (δ)	0.221**	0.044	0.090	0.114***
	(0.093)	(0.075)	(0.083)	(0.036)
Controls	Yes	Yes	Yes	Yes
Observations	488	489	488	488
R^2	0.235	0.421	0.318	0.745

Source: Authors' analysis based on data from the Young Lives Study (YLS) in Ethiopia. Data on foundational cognitive skills (FCS) were obtained during the fourth YLS survey round in 2013.
Note: Analysis performed on the bottom 80 percent of the 2006 wealth index distribution. The table reports the OLS estimates with standard errors (reported in parentheses) clustered at community level. LTM = long-term memory, IC = inhibitory control, WM = working memory, IL = implicit learning. RACER outcomes are standardized using the means and standard deviations of the control group. Each coefficient comes from a different estimation of equation (1) for each FCS outcome. All estimations include community fixed effects, and control for the age and gender of the child, the child's main language, religion, and ethnicity, the socio-economic status of the household, the household size, the household head's gender and education, whether the household owned their house and the land on which the house was built, the household's food expenditure, whether the household has access to a school, whether the household has an influential connection, the number of people the family can rely on for financial need, and whether the caregiver believes that a child should leave school for work if needed; p-values are calculated using wild bootstrap standard errors (clustered at the community level) derived from running 1,000 replications.
***Significant at 1 percent. **Significant at 5 percent. *Significant at 10 percent.

Table S.1.9. Pre-program Balance, by 2013 PSNP Graduation Status

	2009 & 2013 PSNP beneficiaries	2009-only PSNP beneficiaries (graduated)
Household size	6.16	6.64**
Head of house schooling grade	1.30	1.40
Lowest wealth tercile	0.48	0.53
Access to electricity	0.13	0.07*
Parent has regular salaried employment	0.03	0.00**
Observations	277	145

Source: Data comes from the Young Lives Study (YLS) in Ethiopia.
Note: All variables are measured at Round 2 (2006). Asterisks reflect p-values for a t-test for differences in means between control households and PSNP households. Wealth terciles are based on the Young Lives wealth index (Briones 2017).
***Significant at 1 percent. **Significant at 5 percent. *Significant at 10 percent.

Table S.1.10. Associations between the PSNP and FCS, By Gender

	(1) LTM	(2) IC	(3) WM	(4) IL
PSNP	0.378***	0.015	0.119	0.107
	(0.116)	(0.106)	(0.074)	(0.074)
PSNP ∗ Male (φ)	−0.295*	−0.060	−0.087	0.027
	(0.130)	(0.123)	(0.138)	(0.075)
Controls	Yes	Yes	Yes	Yes
Observations	608	609	608	608
R^2	0.253	0.427	0.305	0.746

Source: Authors' analysis based on data from the Young Lives Study (YLS) in Ethiopia. Data on foundational cognitive skills (FCS) were obtained during the fourth YLS survey round in 2013.
Note: The table reports the OLS estimates with standard errors (reported in parentheses) clustered at community level. Each coefficient comes from a different estimation of equation (2) for each FCS outcome. LTM = long-term memory, IC = inhibitory control, WM = working memory, IL = implicit learning. RACER outcomes are standardized using the means and standard deviations of the control group. All estimations include community fixed effects, and control for the age and gender of the child, the child's main language, religion, and ethnicity, the socioeconomic status of the household, the household size, the household head's gender and education, whether the household owned their house and the land on which the house was built, the household's food expenditure, whether the household has access to a school, whether the household has an influential connection, the number of people the family can rely on for financial need, and whether the caregiver believes that a child should leave school for work if needed; p-values are calculated using wild bootstrap standard errors (clustered at the community level) derived from running 1,000 replications. ***Significant at 1 percent. **Significant at 5 percent. *Significant at 10 percent.

Table S.1.11. Associations between the PSNP and LTM, Separately by Gender

	**Males**	**Females**
PSNP (δ)	0.088	0.486**
	(0.099)	(0.174)
Controls	Yes	Yes
Observations	330	278
R^2	0.331	0.295

Source: Authors' analysis based on data from the Young Lives Study (YLS) in Ethiopia. Data on foundational cognitive skills (FCS) were obtained during the fourth YLS survey round in 2013.

Note: The table reports the coefficients from a separate estimation, by gender, of equation (1) using Long-term Memory (LTM) as the outcome. Standard errors (reported in parentheses) are clustered at community level. LTM outcomes are standardized using the means and standard deviations of the control group. All estimations include community fixed effects and control for the age of the child, the child's main language, religion, and ethnicity, the socio-economic status of the household, the household size, the household head's gender and education, whether the household owned their house and the land on which the house was built, the household's food expenditure, whether the household has access to a school, whether the household has an influential connection, the number of people the family can rely on for financial need, and whether the caregiver believes that a child should leave school for work if needed; p-values are calculated using wild bootstrap standard errors (clustered at the community level) derived from running 1,000 replications.

***Significant at 1 percent. **Significant at 5 percent. *Significant at 10 percent.

Table S.1.12. Full Distribution of Time-Use in 2006

**Hours**	**Unpaid labor**	**Household responsibilities**
0	271	205
1	27	42
2	28	61
3	17	28
4	34	35
5	6	6
6	11	15
7	0	6
8	8	5
9	0	0
10	2	2
11	0	0
12	1	0
Observations	405	405

Source: Data from the second round of the Young Lives Study (YLS) in Ethiopia.

Note: Hours refer to the number of hours a respondent spent in each category during a typical day (from Monday to Friday) in the week before the survey. "Unpaid labor" refers to time in tasks on the family farm or business. "Household responsibilities" refers to time spent caring for others or in domestic tasks (fetching water, firewood, cleaning, cooking, washing, shopping, etc.). Time-use information was only asked about children aged five and over in 2006.

Table S.1.13. Heterogenous Associations According to 2006 Time-Use, Public Works Households Only

	Unpaid labor<br>(1)	Household responsibilities<br>(2)
**Panel A. Long-term memory (LTM)**		
PSNP	0.151	0.309**
	(0.129)	(0.166)
PSNP * Reported any hours	0.098	−0.210
	(0.245)	(0.149)
PSNP among those who reported any hours	0.249	0.099
	(0.265)	(0.154)
Observations	367	367
R^2	0.259	0.263
**Panel B. Implicit learning (IL)**		
PSNP	0.037	0.153**
	(0.072)	(0.070)
PSNP * Reported any hours	0.284*	−0.024
	(0.155)	(0.075)
PSNP among those who reported any hours	0.321**	0.129
	(0.127)	(0.075)
Observations	367	367
R^2	0.766	0.762

Source: Authors' analysis based on data from the Young Lives Study (YLS) in Ethiopia. Data on foundational cognitive skills (FCS) were obtained during the fourth YLS survey round in 2013. Data on pre-program time use is from the second YLS survey round in 2006.

Note: PSNP households only include those benefitting from the public works program (not those receiving direct support). The table reports OLS estimates with standard errors (reported in parentheses) clustered at community level. Panel A reports the heterogeneous associations between participation in the Productive Safety Net Programme (PSNP) and LTM according to pre-program time-use; Panel B reports the heterogeneous associations between participation in the PSNP and IL according to pre-program time-use. For both LTM and IL, equation (2) is estimated with each time-use binary variable. RACER outcomes are standardized using the mean and standard deviation of the control group. All estimations include community fixed effects, and control for the age and gender of the child, the child's main language, religion, and ethnicity, the socioeconomic status of the household, the household size, the household head's gender and education, whether the household owned their house and the land on which the house was built, the household's food expenditure, whether the household has access to a school, whether the household has an influential connection, the number of people the family can rely on for financial need, and whether the caregiver believes that a child should leave school for work if needed; *p*-values are calculated using wild bootstrap standard errors (clustered at the community level) derived from running 1,000 replications. "Reported any hours" takes the value of 1 if the participant reported any hours in the relevant time use category in 2006, and 0 otherwise. "PSNP among those who reported any hours" coefficient reflects the association between the PSNP and the FCS of children who reported spending at least some time in each time-use category in 2006. It is calculated as the linear combination of the PSNP and PSNP * Reported any hours regression coefficients. The sample size in this table is slightly reduced, as time-use information was not collected on children under five in 2006.

***Significant at 1 percent. **Significant at 5 percent. *Significant at 10 percent.

Table S.1.14. Association between the PSNP and Body Mass Index

	(1)<br>Body Mass Index (2013)
PSNP	0.243***
	(0.072)
Controls	Yes
Observations	609
R^2	0.360

Source: Authors' analysis based on data from the Young Lives Study (YLS) in Ethiopia.

Note: The table reports the OLS estimate with standard errors (reported in parentheses) clustered at community level. Estimation controls for pre-program (2006) BMI, community fixed effects, the age and gender of the child, the child's main language, religion, and ethnicity, the socioeconomic status of the household, the household size, the household head's gender and education, whether the household owned their house and the land on which the house was built, the household's food expenditure, whether the household has access to a school, whether the household has an influential connection, the number of people the family can rely on for financial need, and whether the caregiver believes that a child should leave school for work if needed; *p*-values are calculated using wild bootstrap standard errors (clustered at the community level) derived from running 1,000 replications.

***Significant at 1 percent. **Significant at 5 percent. *Significant at 10 percent.

Table S.1.15. Heterogenous Associations According to Early-Life Rainfall Shocks, Using 2.5 SPI Cut-Off

	In-utero (1)	Year 1 (2)	Year 2 (3)
**Panel A: Long-term memory**			
PSNP	0.134**	0.093	0.009
	(0.063)	(0.058)	(0.062)
Rainfall shock	−0.140	−0.298	−0.080
	(0.542)	(0.097)	(0.323)
PSNP * Rainfall shock	0.289	0.352	0.326**
	(0.277)	(0.145)	(0.114)
Observations	512	512	512
Controls	Yes	Yes	Yes
R^2	0.224	0.226	0.226
**Panel B: Implicit learning**			
PSNP	0.114**	0.112**	0.133**
	(0.057)	(0.048)	(0.065)
Rainfall shock	−0.010	0.152	−0.304
	(0.069)	(0.107)	(0.174)
PSNP * Rainfall shock	−0.040	−0.016	−0.052
	(0.070)	(0.136)	(0.126)
Observations	512	512	512
Controls	Yes	Yes	Yes
R^2	0.740	0.740	0.740

Source: Authors' analysis based on data from the Young Lives Study (YLS) in Ethiopia and rainfall data from the University of Delaware (Matsuura and Willmott 2018). Data on foundational cognitive skills (FCS) were obtained during the fourth YLS survey round in 2013.

Note: Analysis is only performed on children who have complete GPS information and did not move communities between conception and Round 2 (~ age five). The table reports the OLS estimates with robust standard errors (reported in parentheses) clustered at community level. LTM = long-term memory, IL = implicit learning. RACER outcomes are standardized using the mean and standard deviation of the control group. All estimations include community fixed effects, and control for the age and gender of the child, the child's main language, religion, and ethnicity, the socioeconomic status of the household, the household size, the household head's gender and education, whether the household owned their house and the land on which the house was built, the household's food expenditure, whether the household has access to a school, whether the household has an influential connection, the number of people the family can rely on for financial need, and whether the caregiver believes that a child should leave school for work if needed; *p*-values for wild bootstrap derived from running 1,000 replications. Wild bootstrap clustered at the community level. "Rainfall shock" takes the value of 1 if the participant experienced at least one month in the in-utero period, first, or second year of life, respectively, where the Standardized Precipitation Index deviated at least 2.5 standard deviations above or below the historical monthly average of the same community. "PSNP among Rainfall shock sample" coefficient reflects the effect of the PSNP among children who experienced at least one rainfall shock in each period. It is calculated as the linear combination of the PSNP and PSNP * Rainfall shock regression coefficients.

***Significant at 1 percent. **Significant at 5 percent. *Significant at 10 percent.

Table S.1.16. Associations between the PSNP and FCS, Controlling for Other Social Protection Programs

	(1) LTM	(2) IC	(3) WM	(4) IL
PSNP (δ)	0.209**	−0.020	0.073	0.127***
	(0.081)	(0.058)	(0.057)	(0.050)
Controls	Yes	Yes	Yes	Yes
	608	609	608	608
Observations				
R^2	0.250	0.429	0.306	0.747

Source: Authors' analysis based on data from the Young Lives Study (YLS) in Ethiopia. Data on foundational cognitive skills (FCS) were obtained during the fourth YLS survey round in 2013.

Note: The table reports OLS estimates from equation (1), controlling for whether, in 2013, the household reported receiving the Health Extension Programme, Emergency Aid, or any other social protection program. All estimations also include community fixed effects, and control for the age and gender of the child, the child's main language, religion, and ethnicity, the socioeconomic status of the household, the household size, the household head's gender and education, whether the household owned their house and the land on which the house was built, the household's food expenditure, whether the household has access to a school, whether the household has an influential connection, the number of people the family can rely on for financial need, and whether the caregiver believes that a child should leave school for work if needed. Robust standard errors (reported in parentheses) are clustered at community level. LTM = long-term memory, IC = inhibitory control, WM = working memory, IL = implicit learning. RACER outcomes are standardized using the means and standard deviations of the control group. Each coefficient comes from a different estimation of equation (1) for each FCS outcome; *p*-values calculated using wild bootstrap standard errors (clustered at the community level) derived from running 1,000 replications.

***Significant at 1 percent. **Significant at 5 percent. *Significant at 10 percent.

Table S.1.17. Association between the PSNP and Time in Educational Activities

	Hours in educational activities (2013)
PSNP (δ)	0.313**
	(0.146)
Observations	405
R^2	0.130

Source: Authors' analysis based on data from the Young Lives Study (YLS) in Ethiopia.

Note: The table reports the coefficient from regressing hours in educational activities in 2013 on participation in the PSNP. Educational activities refers to both time in school and time studying at home. Standard errors (reported in parentheses) are clustered at community level. The estimation includes community fixed effects and controls for hours in education in 2006, the age and gender of the child, the child's main language and ethnicity, the socioeconomic status of the household (as measured through the YLS wealth index), and the household size; *p*-values are calculated using wild bootstrap standard errors (clustered at the community level) derived from running 1,000 replications.

***Significant at 1 percent. **Significant at 5 percent. *Significant at 10 percent.

References

Behrman, J.R., K. Briones, S. Cueto, M. Favara, R. Freund, A. Hittmeyer, J. Lopez, N.P. Navarro, A. Sánchez, D. Scott, M.A. Sheridan, and T. Woldehanna. 2022. "Measuring Foundational Cognitive Skills in Young Lives Using RACER." Technical Note 54. Young Lives. Oxford, UK.

Briones, K. 2017. "How Many Rooms Are There in Your House? Constructing the Young Lives Wealth Index." *Technical Note* 43. Young Lives: 1–18.

Matsuura, K., and C.J. Willmott. 2018. Terrestrial Air Temperature and Precipitation: 1900–2017 Gridded Monthly Time Series (V 5.01).NOAA PSL, Boulder, Colorado, USA. Accessed at https://psl.noaa.gov.

Oster, E. 2019. "Unobservable Selection and Coefficient Stability: Theory and Evidence." *Journal of Business and Economic Statistics* 37(2): 187–204.

WHO Multicentre Growth Reference Study Group. 2006. "Assessment of Differences in Linear Growth among Populations in the WHO Multicentre Growth Reference Study." *Acta Paediatrica Supplement* 450: 56–65.

© The Author(s) 2023. Published by Oxford University Press on behalf of the International Bank for Reconstruction and Development/THE WORLD BANK. This is an Open Access article distributed under the terms of the Creative Commons Attribution License (https://creativecommons.org/licenses/by/4.0/), which permits unrestricted reuse, distribution, and reproduction in any medium, provided the original work is properly cited.
